# Interventions pathways to reduce tuberculosis-related stigma: a literature review and conceptual framework

**DOI:** 10.1186/s40249-022-01021-8

**Published:** 2022-09-23

**Authors:** Charlotte Nuttall, Ahmad Fuady, Holly Nuttall, Kritika Dixit, Muchtaruddin Mansyur, Tom Wingfield

**Affiliations:** 1 Department of Clinical Infection, Microbiology and Immunology, University of Liverpool, Liverpool, UK; 2 Department of Community Medicine, Faculty of Medicine, Universitas Indonesia, 10310 Jakarta, Indonesia; 3 Department of Public Health, Erasmus MC University Medical Center Rotterdam, 3015GD Rotterdam, The Netherlands; 4 Primary Health Care Research and Innovation Center, Indonesian Medical Education and Research Institute, Faculty of Medicine Universitas Indonesia, 10430 Jakarta, Indonesia; 5 Social Medicine, Infectious Diseases, and Migration (SIM) Group, Department of Public Health Sciences, Karolinska Institute, 10653 Stockholm, Sweden; 6 Birat Nepal Medical Trust, Lazimpat Road, Lazimpat, Kathmandu, 44600 Nepal; 7 Departments of International Public Health and Clinical Sciences, Liverpool School of Tropical Medicine, Liverpool, L3 5QA UK; 8 Tropical and Infectious Disease Unit, Liverpool University Hospitals NHS Foundation Trust, Liverpool, L7 8XP UK

**Keywords:** Tuberculosis, Stigma, Intervention, Stigma measurement tool

## Abstract

**Background:**

Prevention of tuberculosis (TB)-related stigma is vital to achieving the World Health Organisation’s End TB Strategy target of eliminating TB. However, the process and impact evaluation of interventions to reduce TB-stigma are limited. This literature review aimed to examine the quality, design, implementation challenges, and successes of TB-stigma intervention studies and create a novel conceptual framework of pathways to TB-stigma reduction.

**Method:**

We searched relevant articles recorded in four scientific databases from 1999 to 2022, using pre-defined inclusion and exclusion criteria, supplemented by the snowball method and complementary grey literature searches. We assessed the quality of studies using the Crowe Critical Appraisal Tool, then reviewed study characteristics, data on stigma measurement tools used, and interventions implemented, and designed a conceptual framework to illustrate the pathways to TB-stigma reduction in the interventions identified.

**Results:**

Of 14,259 articles identified, eleven met inclusion criteria, of which three were high quality. TB-stigma reduction interventions consisted mainly of education and psychosocial support targeted predominantly toward three key populations: people with TB, healthcare workers, and the public. No psychosocial interventions for people with TB set TB-stigma reduction as their primary or co-primary aim. Eight studies on healthcare workers and the public reported a decrease in TB-stigma attributed to the interventions. Despite the benefits, the interventions were limited by a dearth of validated stigma measurement tools. Three of eight studies with quantitative stigma measurement questionnaires had not been previously validated among people with TB. No qualitative studies used previously validated methods or tools to qualitatively evaluate stigma. On the basis of these findings, we generated a conceptual framework that mapped the population targeted, interventions delivered, and their potential effects on reducing TB-stigma towards and experienced by people with TB and healthcare workers involved in TB care.

**Conclusions:**

Interpretation of the limited evidence on interventions to reduce TB-stigma is hampered by the heterogeneity of stigma measurement tools, intervention design, and outcome measures. Our novel conceptual framework will support mapping of the pathways to impacts of TB-stigma reduction interventions.

**Graphical Abstract:**

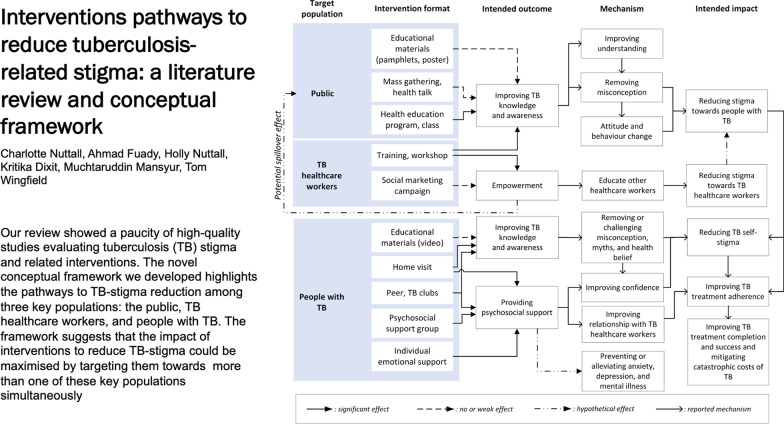

**Supplementary Information:**

The online version contains supplementary material available at 10.1186/s40249-022-01021-8.

## Background

Stigma experienced by people with or affected by tuberculosis (TB)—henceforth termed TB-stigma—remains one of the major challenges in TB control [1, 2]. TB-stigma has been shown to delay health-seeking behaviour [3]. This challenge has been aggravated by the COVID-19 pandemic which has restricted access to healthcare, reduced the number of people notified with TB, and been associated with an increase in TB mortality [1, 4, 5]. TB-stigma has also been shown to reduce treatment compliance, and negatively impact on TB treatment outcomes [6, 7]. The prevalence of TB-stigma varies geographically and, in specific subpopulations, has been estimated to affect up to 80% of people with TB [8, 9]. Therefore, in the context of TB control, TB-stigma is one of the major social determinants of health and contributes to compounding health inequalities [1, 10, 11].

For these reasons, the Global Fund and UN high-level meeting highlighted TB-Stigma as one of the most significant barriers to reaching the World Health Organization (WHO) End TB goal of eliminating TB by 2050 and called on the international community to “promote and support an end to stigma and all forms of discrimination” [12–14]. Despite this, few resources have been mobilised to address this issue [15]. In part, this is due to inherent difficulties in the identification and measurement of TB-Stigma and the complexity and limited evidence base relating to stigma-reduction interventions [16].

Measuring stigma is vital to understand its determinants, prevalence and assess the effectiveness of stigma-reduction interventions [17]. Multiple scales and tools exist that assess health-related stigma [18]. To be robust and reliable, these tools should have been validated in the community or population in which they are to be used and then refined to ensure they are accurate, specific, and reliable. In 2018, the KNCV Tuberculosis Foundation created a TB-Stigma Handbook, which provides examples of how the limited available set of existing tools can best be applied to measure and evaluate stigma [19]. However, most studies to date have used either disparate, invalidated tools or solely qualitative measures of stigma. This has made it difficult to broaden our understanding of the determinants and consequences of TB-Stigma [15, 20] (Box 1).

In addition, despite recognition of the global importance of TB-stigma, there has been limited critical appraisal in the literature of the few existing interventions aimed at reducing TB-stigma. The single related systematic review on TB-Stigma by Sommerland et al. focused on the effectiveness of stigma-reduction interventions [17]. Measuring and reducing TB-Stigma is complex. It involves interrelated, heterogeneous system structures, and multiple approaches from the individual to societal level [16]. Therefore, it is critical to evaluate not only the scale but also the challenges and successes in the design and implementation processes of interventions to reduce TB-stigma. These evaluations will help identify the weaknesses in current TB-stigma intervention design and delivery in order to refine these interventions for future implementation and scale-up.

We reviewed studies reporting interventions to reduce TB-Stigma. The review appraised the study design and stigma measurement tools, identified their challenges and successes, and evaluated their pathways to impact on TB-Stigma. We then developed a conceptual framework for the TB-Stigma reduction pathway to support researchers to successfully design and deliver impactful TB-stigma reduction interventions.

Box 1 Types of TB-stigma [18] **Enacted (or experienced) stigma** encompasses the range of behaviours directly experienced by a person with TB.

**Anticipated** **stigma** is the expectation and fear of discrimination and behaviour of others towards a person if they are diagnosed and/or unwell with TB, which has an impact on health-seeking behaviour, whether enacted stigma occurs or not.

**Internalised** **(or self)** **stigma** is when those diagnosed and/or unwell with TB may accept a negative stereotype about people with TB and potentially act in a way that endorses this stereotype.

**Secondary** **or external stigma** is negative attitude towards family members, caregivers, friends, or TB healthcare workers because they are associated with, live with, or have close contact with people with TB.

## Methods

This study was a systematic literature review. A preliminary scoping search was conducted to ensure that all relevant key terms were identified, and the final search strategy refined.

### Search terms and management of search results

The following search terms were used within four databases (CINAHL Complete, Medline Complete, Global Health and PubMed): (TB OR Tubercul* OR “Mycobacterium tuberculosis infections”) AND (stigma* OR discrimin* OR “social stigma” OR barrier* OR attitude* OR “social discrimination” OR marginalisation OR “psychosocial impact” OR “socioeconomic impact” OR shame OR “social isolation” OR “social inclusion” OR prejudice OR perception OR “self-esteem”) AND (interven* OR strateg* OR pathway* OR education OR “psychosocial intervention*” OR “psychoemotional intervention*” OR “socioeconomic intervention*” OR “social support” OR “patient support” OR “training workshop*” OR “counselling”) were searched. In addition, the “snowballing” method of reference tracking and searches of Google Scholar and the WHO database for grey literature were used to identify additional articles that may have been overlooked by the initial search strategy. Searches were limited to December 31, 2021. Citations of the articles identified from the searches were exported into Endnote X9 (Camelot UK Bidco Limited/Clarivate, UK). Duplicates were then identified and removed using the duplicates tool in Endnote X9. The titles and abstracts of the remaining articles were read through and screened for relevance independently by three reviewers (CN, HN, AF). Where there were unresolved disagreements, a fourth senior reviewer finalized screening for inclusion or exclusion (TW). We applied the inclusion and exclusion criteria, documented the reasons for article exclusion, and identified the relevant articles for full-text review. Finally, supplemental manual review of the reference lists of the selected full-text articles was performed to identify any further articles for inclusion.

### Selection criteria

Eligible studies included those that reported the implementation and evaluation of TB-stigma reduction interventions amongst people with TB and their households, healthcare workers, and the general public. Included study designs were intervention studies with randomised controlled trials, non-randomised controlled trials, quasi-experimental studies, mixed-methods studies, qualitative studies, cohort studies, case-control studies, and cross-sectional studies. The review was restricted to articles that were written in English. Articles were excluded if they did not report measurement of stigma.

### Critical appraisal

Critical appraisal was undertaken by three reviewers (CN, HN, AF) using the “Crowe Critical Appraisal Tool” (CCAT), Version 1.4, to determine the quality of each study [21]. The CCAT was selected as it has been proven to be reliable and valid for the analysis of multiple studies of heterogenous design and implementation approaches, and can reduce rater bias [22]. To further reduce researcher bias, each individual assessment was cross-checked. A fourth reviewer (TW) resolved any discrepancies. A priori, and in line with published guidance [21], a pragmatic decision was taken by the study team that articles with a CCAT score between 75% and 100% would be deemed high quality, 50% and 74% moderate, and below 50% to be low quality.

### Data extraction and synthesis

Data on country and region of intervention, target population, type of stigma studied, the scale or tool used to assess stigma, intervention activities, challenges and successes of intervention, the impact of intervention, and reported changes to TB practice and policy were collated and tabulated. Further information on the intervention including format, content, outcomes (both reported and intended if different) and detail on how the intervention reduced TB-stigma (theory explicitly stated in the main text or implied in objectives or methods) were also tabulated. Qualitative details were lifted directly from the text and copied into the data-extraction table. The articles were read carefully for similar and recurring themes and concepts. The concepts were then organised to determine any contradictory concepts, which were then removed. A conceptual framework was then created to organise the variables and concepts perceived by the research team to contribute to the pathways by which an intervention successfully reduced TB-stigma. The intention of the novel conceptual framework was to support researchers to design, develop, and implement a successful and sustainable stigma-reduction intervention in current and future studies [23]. With respect to stigma measurement tools and scales, these were evaluated through collection of data including: the tool or scale used; implementation methods; methods to reduce bias and ensure validity; internal and external validation and piloting prior to use; comparison of stigma scores before and after the intervention or between study groups; types of stigma assessed; whether the tool was adapted from a previously validated tool; and the described limitations of the tool. Any required data that was missing from the published papers was collected by directly contacting the corresponding author of the paper.

## Results

**Fig. 1:**
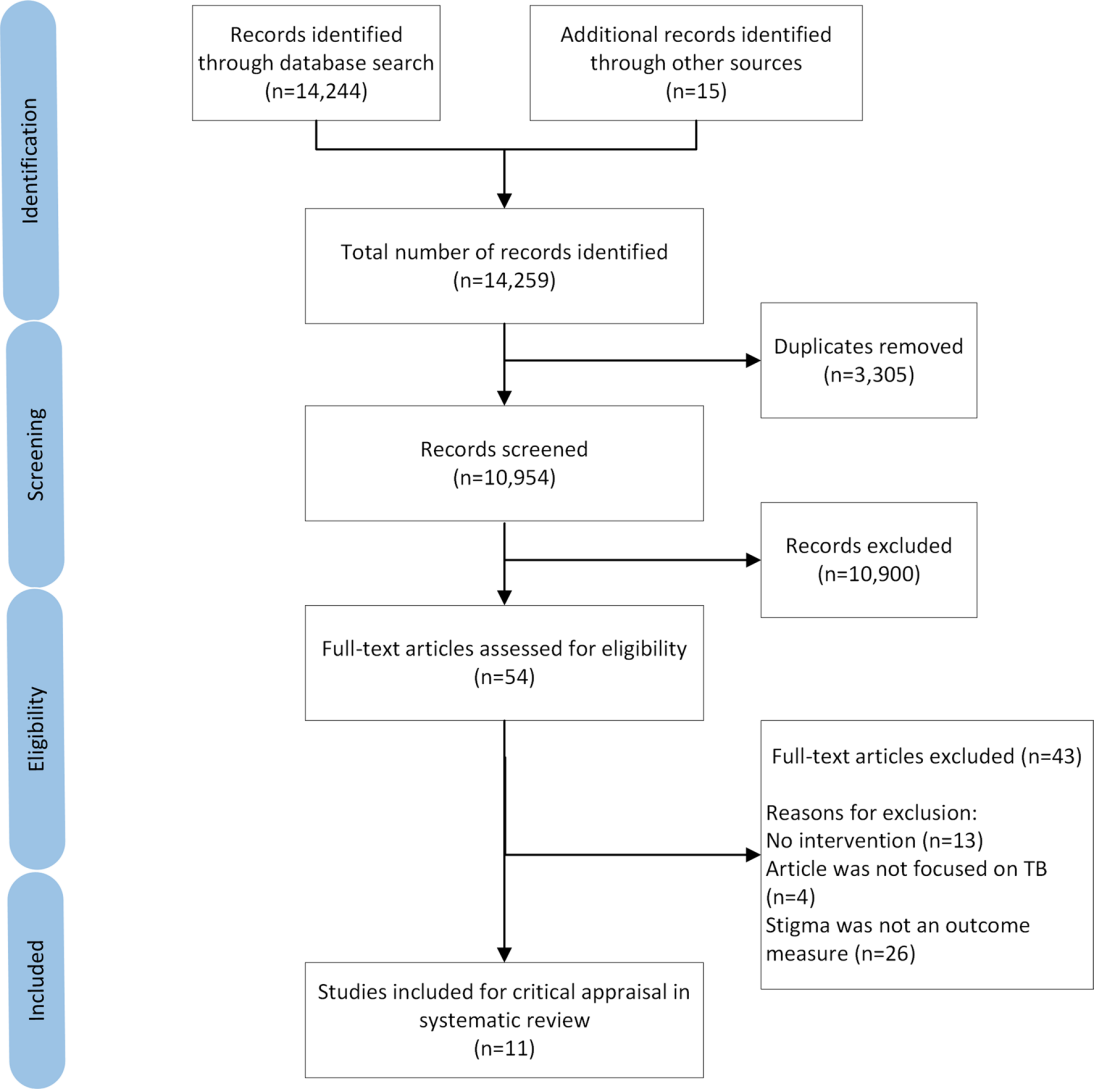
PRISMA flow diagram of study identification, screening, and inclusion in review. Other sources of articles refer to those identified through the Stop TB Partnership website, KNCV Tuberculosis foundation database, and the snowball method

The search yielded 14,244 articles with 15 further articles identified from other sources including grey literature. After removal of duplicate articles, 10,954 were screened, 54 of which met the study inclusion criteria. Following the full-text eligibility assessment, 43 further articles were excluded (Additional file 1). The remaining 11 articles were included for critical appraisal in the systematic review (Fig. 1).

### Quality assessment

The median CCAT score for quality of studies was 24/40 (range 15–38) (Additional file 1). Three studies were classified as high quality [24–26]. The predominant reasons for lower quality scoring were lack of details relating to methods and study protocols including sampling frames and ethical approval.

### Study characteristics

There was marked heterogeneity in the study characteristics in terms of study aims, designs, population and region/sites, and type of stigma measured (Table 1). The studies were conducted in low- (*n* = 1) [27], middle- (*n* = 9) [24–26, 28–33], and high-income (*n* = 1) countries [34]. Two studies were conducted in the same country (Peru) [30, 31], and these studies were linked with some overlap of study team members and co-authors. The studies were targeted at a variety of different populations including people with TB and MDR-TB and their households (*n* = 5), HCWs (*n* = 3), and the public (*n* = 3).

**Table 1:** Characteristics of TB-stigma intervention studies

Authors	Region and country	Study design and intervention	Type of stigma	Aims	Sample and study population
Population: People with TB					
Macq et al. 2008 [28]	Rural Nicaragua	Quasi-experimental TB clubs: group meeting between people with TB Patient-centred home visits: home visits and discussion by HCWs	Internalised stigma	(1) Reduction of internalised stigma (2) Improving TB treatment outcomes	268 newly diagnosed people with TB (122 intervention, 146 control)
Demissie, Getahun and Lindtjørn, 2003 [29]	Rural Northern Ethiopia	Non-randomized controlled trial TB clubs: group visit to clinic, regular weekly meeting, and ad-hoc meeting on holidays or market days	Internalised and anticipated stigma	(1) Improving treatment compliance (2) Assessing societal changes including internalised and anticipated stigma	128 people with TB (64 intervention group, 64 in comparison group)
Acha et al. 2007 [30]	Peru	Non-randomized trial in eight groups Psychosocial support intervention: Support groups, recreational excursions, symbolic celebrations, and periodic family workshops	Internalised stigma	(1) Improving treatment completion (2) Reducing psychosocial impact of TB including internalised stigma	285 people with MDR-TB
Wilson et al. 2016 [33]	El Salvador	Pilot intervention study Educational videography: basic TB information including transmission, TB diagnosis and treatment, common misconceptions and misunderstandings related to TB, and testimonials of people with TB	Anticipated and enacted stigma	(1) Assessing the feasibility of intervention implementation (2) Improving the understanding of TB among people with TB and their family members, including anticipated stigma	1916 people with TB and family members watched the video 15 people with TB and their families were evaluated
Chalco et al. 2006 [31]	Lima, Peru	Program evaluation, qualitative Community nursing: healthcare visit, home visit, and support group therapy	Internalised stigma	Identifying forms and means of emotional support by nurses	Intervention for people receiving MDR TB treatment from 1996–2004 Feedback evaluation of seven nurses
Bond et al. 2017 [26]	Zambia and South Africa	Cluster randomised trial: Household counselling intervention	Internalised and enacted stigma	(1) Developing TB-Stigma items (2) Evaluating the stigma after intervention	1826 people with TB and 1235 household members of people with TB from different 24 communities
Population: Healthcare workers					
Wu et al. 2009 [34]	Taiwan, China	Quasi-experimental TB training course: TB education, information of TB epidemiology, skills for DOTS execution, de-stigmatisation, and human rights	Anticipated stigma	(1) Increasing TB knowledge (2) Reducing anticipated TB-Stigma	1279 HCWs
Sommerland et al. 2020 [24]	South Africa	Cluster randomised controlled trial Workshop: training and social marketing campaign	External (secondary) stigma	Reducing HIV and TB enacted and secondary stigma	652 HCWs in eight hospitals (367 in intervention group, 285 in control group)
Population: Public					
Croft and Croft, 1999 [27]	Panchagar district, Bangladesh	Program evaluation, case control Health education programme: mass information program using flipchart, loudspeaker, and slide stories of successful treatment	Anticipated stigma	Improving knowledge, attitude, and practice on leprosy and TB Stigma was included in attitude measurement	Intervention to two Unions/area (each with approximately 20,000 population) Evaluation in 100 adults
Balogun et al. 2015 [32]	Idi Araba region, Southwest Nigeria	Quasi-experimental Organized community volunteer programs: health talk, one-on-one discussion, educational pamphlets, and street rally	Anticipated stigma	Improving TB knowledge, attitude, and practice Stigma was included in knowledge and attitude measurements	Intervention to community with ± 42,000 population Evaluation in 252 adults aged more than 18 years old
Idris et al. 2020 [25]	Kelantan, Malaysia	Non-randomised controlled trial Education program: lecture, quiz session, small group discussions, poster exhibition, and four booklets on TB Control: information on adolescent health and hygiene	Anticipated stigma	(1) Increasing knowledge, attitudes, and preventive behaviours towards TB (2) Reducing stigma	236 secondary high school students (118 in intervention group, 118 in control group)

*DOTS* directly observed treatment, short-course, *HCW* healthcare workers, *HIV* human immunodeficiency virus, *MDR-TB* multidrug-resistant TB, *TB* tuberculosis

### Study population, aims and intervention

Six studies applied interventions targeted towards people with TB. Five of the studies aimed to improve TB treatment compliance and completion through psychosocial support interventions, which were TB clubs or support groups (*n* = 3) [28–30], nurse support (*n* = 1) [31], and household counselling (*n* = 1) [26], while one study focused on improving TB knowledge [33].

TB clubs involved group meetings of people diagnosed with TB to discuss their experiences and provide mutual support to encourage each other through their illness and treatment. Other studies initiated patient-centred home visits by HCWs to complement the TB clubs [30], provided individualised emotional support from community nurses who informed and educated people with TB and their households about TB [31], and implemented a household counselling intervention delivered by nurses and trained counsellors [26]. All of the studies tailored towards people with TB captured stigma related to being diagnosed with TB. The assessment focused on measuring enacted and internalised stigma and, where possible, the influence of such stigma on TB treatment success rates. Two of the studies also involved family members of people with TB: one to evaluate stigma [26] and another to evaluate people with TB and their family members’ TB knowledge following delivery of educational videos while waiting at TB outpatient clinic appointments [33].

Two studies evaluated stigma among HCWs using workshops focused on distinct aspects of stigma [24, 34]. One delivered nationwide TB training workshops to educate HCWs on TB, stigma and human rights to improve knowledge on TB and reduce TB-stigma towards people with TB [34]. In another, there was a focus on healthcare workers who were themselves stigmatised by other HCWs [24]. This study measured external or secondary stigma, in which HCWs experience negative attitudes or rejection because of the care they have given to people with TB.

Three studies assessed anticipated TB-Stigma among the public: two in an adult population and one in an adolescent population. All the studies measured anticipated TB-stigma using before-after intervention designs. Two studies applied health education programs in the community (mass information programs and health promotion at mass gatherings) [27, 32]. Another study delivered training to students and evaluated whether the training reduced their levels of anticipated TB-stigma [25].

### Stigma measurement tools

Eight studies used quantitative questionnaires to measure stigma (Table 2) [24–28, 32–34]. The format of the questionnaires to measure stigma varied widely including the number of questions asked (range 3–14 questions). Three of the questionnaires were adapted from tools that were not specific to any particular disease and had been previously validated but not among people with TB [28, 29, 34]. For example, Macq et al*.* adapted their questionnaire from the Boyd Ritsher Mental Illness stigma scale and pre-tested it 2 years before the intervention study to improve its internal validity [28]. One study piloted the tools in six different communities (four Zambian and two South African) with six different languages (Nyanja, Bemba, Tonga, isiXhosa, Afrikaans and English) [26]. Four other studies piloted their questionnaires in a single population each [24, 25, 27, 28, 35].

**Table 2:** TB-stigma measurement tools

References	Scale/tool	Questionnaire administration	Ensuring bias reduction and validity	Internal and external validation prior the study	Intervention evaluation on stigma reduction	Type of stigma assessed	Limitations to the tool
People with TB							
Macq et al. 2008 [28]	10 statements with Likert Scale responses from 1 (completely disagree) to 5 (completely agree)	Self-applied	Conducted outside the healthcare facility to decrease likelihood of influence of HCWs on survey responses	Piloted for internal validity Adapted previously validated Boyd Ritsher mental illness stigma scale	Scores taken after 15 days and 2 months	Internalised stigma	Self-applied. Adapted from a validated questionnaire that was not specific to TB
Wilson et al. 2016 [33]	Questionnaire with six items	Questions were asked by HCWs	Questionnaire was supplied by the Ministry of Health, so questions were not selected under the influence of researcher bias	Information not given	Before and 1 month after watching the educational video	Anticipated and enacted stigma	Pre- and post-video data were not collected uniformly in the early stages of the study. There was no study coordinator which meant that there were inconsistencies in using the tool
Bond et al. 2017 [26]	Questionnaire with 14 TB-specific items: four questions towards TB-affected household members and 10 towards people with TB	Administered using paper copies by a research assistant and nurse/counsellor	The questionnaire was administered by the same pair of researchers at two time points to ensure consistency	Tool was piloted in six different communities (four in Zambia and four in South Africa)	Before the study and 18 months later	Internalised and enacted stigma	Research assistants were not trained specifically in TB-Stigma which could have affected how questions were asked and interpreted by interviewers
Population: Healthcare Workers							
Wu et al. 2009 [34]	Structured 8-item questionnaire with 5-point Likert Scale (1–extremely unimportant to 5—extremely important)	Participants completed the questionnaire immediately before and after training	Information not given	Used the Attribution Questionnaire – Short forms- 9 items (AQ-S8). No information on validation	Before and after intervention	Anticipated stigma	Adapted from a validated questionnaire that was not specific to TB
Sommerland et al. 2020 [24]	Respondents’ external TB-Stigma (TB-REXT): three items capturing stigmatising attitudes towards colleagues with HIV or TB Other co-workers’ external TB-Stigma (TB-OEXT): five items of *perception* the general attitudes of other co-workers towards co-workers with HIV or TB	Self-administered questionnaire	The questionnaire was very short and easy to be filled	Questionnaire was previously validated and resulted in good reliability (Wouters et al. 2017). It was again tested for its reliability during the study	Baseline (2016) and follow-up (2018)	Enacted and secondary stigma	Both questionnaires were too short; resulting in lower reliability than previous measures of reliability when the questionnaire was first validated (2017)
Population: Public							
Croft and Croft 1999 [27]	Questionnaire with five items and responses limited to “yes”, “no” or “don’t know”	House to house questioning by interviewer. Head of the house usually answered with details added from family members	Questionnaire was deliberately short to ensure it could be completed quickly	Tool had been piloted for internal validity	No	Anticipated stigma	No previous assessments of the tool have been carried out so comparison could not be made
Balogun et al. 2015 [32]	Questionnaire on personal characteristics, living conditions, TB attitudes and care seeking behaviours, TB attitudes and stigma, TB information and preventive practices	Pre-intervention and repeated 6 months post-intervention	Interviewer-administered questionnaires were used, and the same interviewers collected data post-intervention	Used WHO Knowledge Attitudes and Perceptions (KAP) survey. No information on validation	Scores taken at the beginning and 6 months after the intervention	Anticipated stigma (knowledge and attitudes) of the general community	Adapted from a validated questionnaire that was not specific to TB
Idris et al. 2020 [25]	Questionnaire adapted from TB Scale Stigma (Van Rie, 2008)	Self-administered	Information not given	Used TB-Stigma Scale (Van Rie, 2008). No information on validation	Before training and 4 weeks after training	Anticipated stigma	The questionnaire could lead to bias and inaccurate responses. No reliability test was applied

*HCW* healthcare workers, *MDR-TB* multidrug-resistant TB, *TB* tuberculosis, *WHO* World Health Organization

Most studies (*n* = 7) applied the tool before and after a stigma-reduction or related intervention with the time period between the first and second application varying from 4 weeks to 18 months [24–26, 28, 32–34]. One quantitative study did not evaluate stigma before the intervention [27]. The study was an evaluation of an extensive mass health education programme that was implemented for 2–3 years in one case study area and compared to another control study area with a limited health education programme.

Four studies used qualitative methods, such as focus group discussions, interviews and observation, to evaluate stigma [24, 29–31]. No studies used previously validated methods or tools to qualitatively evaluate stigma. However, the qualitative approaches focused less on measurement of stigma and more on exploring how people with TB attempted to combat the stigma perceived by themselves [29] or by other people [30], and how HCWs who work with people with TB struggled to deal with stigmatisation from other HCWs [24].

### Challenges, successes, and outcomes

Implementation and delivery challenges and process indicators such as fidelity, acceptability, and feasibility were infrequently measured or reported in the studies. One study caused a positive change to national practice with the production of a manual to expand the intervention to a wider population [28]. Three studies reported that a success of the intervention was that sustainable changes had been made within the study site communities [26, 29, 33]. However, there was no objective way to measure or verify these changes from the data presented within the study articles.

Four of the studies explicitly stated that their intervention was limited by geographical challenges including some populations not being reached by the intervention [25, 27, 28, 32]. This limited the external validity of the data. Three studies mentioned challenges concerning maintenance and sustainability of the programmes, including identifying participants to take part in the intervention and motivating people to continue engaging with the intervention [30, 31, 34]. Another study mentioned that inviting all HCWs to participate in a workshop about TB was problematic because hospitals were busy and understaffed [24]. The intervention itself was also challenged by issues relating to professional rank, position, and social status of different HCWs, which was perceived as limiting open discussion about the optimal ways to address stigma between HCWs (Table 3).

**Table 3:** Pathways to impact of TB-stigma interventions

References	Intervention format	Outcomes	Challenges of the intervention	Reported pathways and impact of the intervention to reduce stigma
Population: people with TB				
Macq et al. 2008 [28]	TB clubs Patient-centred home visits	Statistically significant difference between internalised stigma scores 2 months following intervention implementation but no difference in scores 15 days following intervention implementation	Different intervention across all nine municipalities Geographical challenges for participants living long distances from others	Self-help groups strengthened relationships between people with TB who took part. Home visits improved the relationship between HCWs and people with TB and were perceived to improve the confidence of people with TB
Demissie et al. 2003 [29]	TB clubs	Anticipated and internalised stigma in people with TB was reduced People with TB were not scared of a diagnosis of TB, removed any myths about TB treatment, communication around TB between community members improved	None identified	Regular meetings meant patients gained support from each other to adhere to treatment and share information about the process of the treatment and TB, which improved communication and reduced isolation. This was shown to reduce anticipated and internalised stigma
Acha et al. 2007 [30]	Psychosocial support groups	Internalised stigma was reduced Improved confidence of participants and improved knowledge of TB	Difficulties in recruiting qualified group facilitators. Logistical difficulties: finding meeting space, attendance, and delays Lack of experience with asking questions associated with social stigma	Support groups improved attitude towards disease and improved confidence, which reduced stigma
Wilson et al. 2016 [33]	Educational video	Improvement in knowledge and understanding of TB and its treatment. Improved patient adherence to treatment. Reduction in fear and stigma of TB	Not effective in busy clinics (video couldn’t be heard). Technical problems: electrical plugs, broken DVD player	Improved ‘curative’ knowledge (e.g., understanding that TB was a curable disease) reduced misconceptions, which reduced stigma
Chalco et al. 2006 [31]	Individualised emotional support from nurses	Stigma was discussed with nurses to fight stigma and nurses educated people with TB and help to reduce social prejudices. Stigma reduction was not evaluated	Very small group of nurses, thus implementation in a larger area could be difficult. Potential for redirecting nurses away from other roles	Nurses educated and informed family and community members. This group then became more knowledgeable about TB which reduced stigma
Bond et al. 2017 [26]	Household counselling intervention	Stigma scale was developed No statistically significant reduction in TB-Stigma and TB prevalence in the community	None identified	The suggested theoretical pathways by which stigma was reduced was that the community felt supported by counselling intervention. This led to empowerment and changes to norms and behaviours, which in turn reduced stigma
Population: Healthcare workers				
Wu et al. 2009 [34]	Nationwide TB training workshops	TB-stigma towards people with TB was reduced significantly. TB knowledge was significantly improved across participants except those with a history of TB. No correlation between increase in TB knowledge and reduction in TB-Stigma	Maintenance of the quality of the intervention across a large, diverse country was difficult	Education workshops caused a shift towards a positive attitude towards TB control which reduced TB-Stigma
Sommerland et al. 2020 [24]	Workshop and social marketing campaign to HCWs	External stigma—measured by questionnaire—was intended to be reduced after intervention	Professional rank, position and social status could intersect with anti- TB-stigma communication. Thus, addressing other power will be useful for further intervention	The intervention was based on Diffusion of Innovations theory. HCWs who were trained were expected to spread knowledge and messages about TB-Stigma in their workplace and to make a substantial impact across the hospitals
Population: public				
Balogun et al. 2015 [32]	10 community volunteers trained to provide community education on TB	Intended outcome was to reduce anticipated stigma. Actual outcome: TB-Stigmatising attitude worsened—more people had a negative attitude towards people with TB following the intervention as misconceptions were not eliminated The other outcome measure, mean knowledge score, improved	Not all ethnic tribes were represented It was challenging to keep community volunteers motivated throughout the intervention period as it was not a paid job	Incomplete education and superficial information on TB in pamphlets led to misconceptions within community which causes changes in TB-stigma
Croft and Croft 1999 [27]	Health education programme	Lower levels of stigma in the union with the enhanced education programme	Boroshoshi Union was visited less as it was not easily accessible. Communities differed significantly thus the education programme might not have been acceptable to all	Education and removal of misconceptions improved community attitudes which reduced stigma
Idris et al. 2020 [25]	Education programme: lecture, quiz, small group discussions, poster, and booklets	Increasing knowledge and practice, and reducing stigma scores, which statistically significant compared to the control group	Evaluation of the education program was within a short period (for weeks). The program would face challenge for scaling up to a wider context with multiple ethnic groups	Increasing TB knowledge to reduce stigma

*DVD* digital video disc, *HCW* healthcare workers, *TB* tuberculosis

### Pathways to impact of TB-stigma interventions

**Fig. 2:**
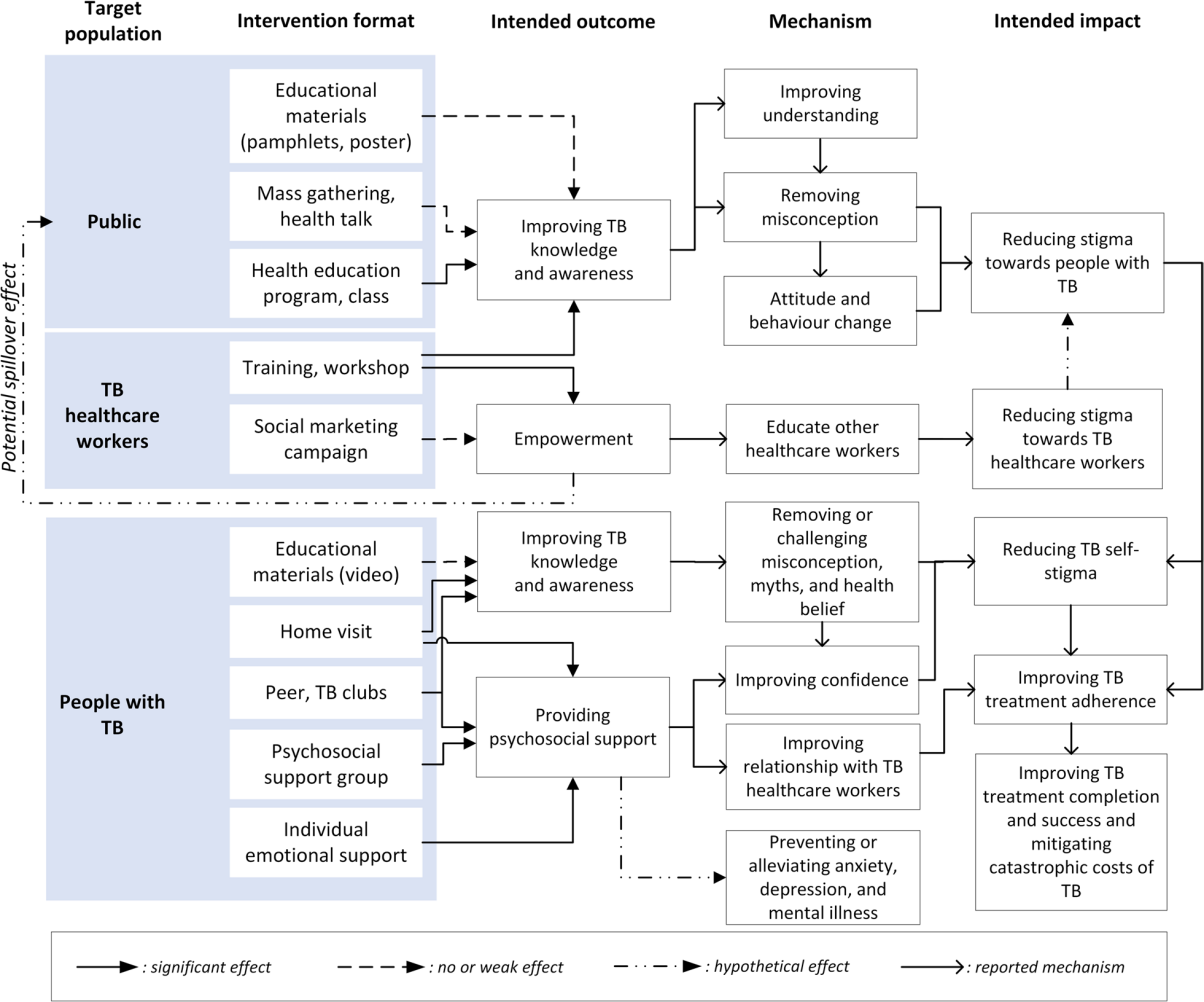
Pathways to impact of TB-stigma interventions

Synthesising learning from the interventions and outcomes, we found distinct pathways to reduce stigma depending on the population targeted by the intervention. We created a novel conceptual framework to illustrate these pathways (Fig. 2).

Among people with TB, stigma is a negative effect of being ill with TB, diagnosed with TB, and being on TB treatment. Stigma towards people with TB can develop in three other populations: the public, TB-related HCWs, and other HCWs. The interventions for people with TB improved TB knowledge, reduced myth and misconception related to TB, increased confidence of people with TB, and thereby reduced internalised stigma experienced by people with TB [28–31, 33]. These effects directly supported people with TB to comply with and complete TB treatment. Another study showed that, although household counselling was not specifically designed to, or found to, reduce TB-Stigma [26], health counsellors can help households manage the consequence of TB-Stigma. The main challenge in providing household counselling, particularly in communities with high levels of TB-stigma, is that the visits themselves may trigger anticipated, internalised, or enacted stigma.

Training to TB HCWs improved the HCWs’ knowledge, attitude, and practice towards people with TB [34], which may contribute to improved TB care. Conversely, our review found that TB-related HCWs were often stigmatised by other HCWs [24]. Training HCWs who care for people with TB to educate other HCWs is likely to support dissemination of knowledge and accurate information about TB-Stigma in their workplace. Although the training failed to reduce external or secondary stigma, there was a potential spill over effect that TB-related HCWs could have used the campaign materials to educate people living in their neighbourhood.

Interventions targeted towards the general public had positive impacts on knowledge, attitudes and practice related to TB as measured by knowledge, attitude, and practice (KAP) and stigma scores [25, 27, 32]. Mass TB education to the public was expected to increase TB knowledge and remove TB misconceptions which could result in improved community attitudes and reduced stigma towards people with TB. However, the evidence found in this review suggested that misconceptions about TB persisted, even worsened, if health education through pamphlets or posters were too short and failed to convey accurate public health messages [32].

## Discussion

This literature review found that, despite the global importance of addressing TB-Stigma, there is a paucity of high-quality studies evaluating interventions to reduce TB-stigma. Intervention design across studies was heterogeneous, but education about TB frequently featured as a core intervention activity. Assessment of the impact of interventions on TB-stigma reduction was limited by a lack of well-validated tools to measure stigma. The novel conceptual framework highlighted that people with TB may experience stigma from three different populations around them: the public, TB HCWs, and other HCWs. The ideal and possibly most synergistic interventions to reduce TB-Stigma would be optimized by delivering interventions targeted towards more than one key populations at the same time.

There has been an increasing awareness of the importance of combatting TB-Stigma in recent years. This study may not have captured stigma-reduction interventions or programs at local, sub-national, or national levels due to such programs not being executed, evaluated, or reported systematically, which makes them difficult to review and compare. Among the limited studies identified, this review found that research on TB-stigma was often hampered by suboptimal design and methods for implementation and evaluation.

This review complements the Sommerland et al*.* paper[17] and extends its findings through a specific focus on the tools used to measure stigma reduction, a qualitative evaluation of the potential reasons underlying the success or failure of the interventions, and the creation of a novel conceptual framework of the pathways to intervention impact. By this approach, this review highlights that most TB-stigma intervention studies used tools that lacked appropriate validation. This finding is consistent with other published studies [36–39]. Using reliable, validated stigma measurement tools and methods is important and helps measure stigma accurately and consistently. It also enables the comparison of impact evaluation of stigma reduction interventions across studies and contexts. There is a list of available tools that can be used and validated [19], including Van Rie’s TB-Stigma Scale [40], one of the most adapted questionnaires to assess stigma [38], which may support researchers and implementers designing TB-stigma reduction programmes in the future.

Using qualitative approaches to measure TB-stigma has both strengths and limitations. This approach cannot precisely assess the reduction of stigma after the intervention. However, it can help explore more profound dimensions of stigma and its impact on people with TB and their households. For example, qualitative studies have been able to elicit key emotional responses to TB-Stigma, including shock, fear of being isolated or abandoned by a spouse, shame related to becoming weak and incapable of working, worry relating to loneliness, and desperation related to thoughts about TB-related death [29, 30]. Therefore, interventions incorporating mixed methods process evaluations would be both prudent and beneficial.

The conceptual framework we have generated can support understanding of the pathways through which interventions successfully reduce stigma and the parties affected. It is notable that interventions rarely have stigma-reduction as a primary aim or objective. Rather, programs often cite stigma reduction as a bridge towards TB treatment compliance, completion, and success. This may be short-sighted: besides improving treatment completion, psychosocial support is also important to prevent or alleviate anxiety, depression, and mental illness, which are well established correlates of being affected by TB [41, 42]. Not having stigma reduction as a primary or even co-primary outcome may have contributed to a lack of focus on using validated instruments to measure stigma. Given that global TB policy strongly recommends interventions to reduce TB-stigma [43, 44], it is vital that appropriately validated tools be used to measure stigma and that reduction of stigma be considered as a key individual-level outcome for people affected by TB.

The framework shows that the interventions on TB HCWs are also critical and may have potential spill over effects to other healthcare workers and the general public. TB HCWs often face secondary stigma and may be at risk of a psychosocial impact of TB themselves, particularly in areas with high co-prevalence of HIV/AIDS and TB [45, 46]. TB-stigma interventions for HCWs can be challenging to implement because they may encounter power structure problems against colleagues with higher professional rank, position, and status [24]. However, HCWs are well placed to convey anti-stigma messages to their surrounding communities and should be empowered to do so.

The framework also emphasises the potential role for synergy across different pathways of TB-stigma reduction to enhance the effectiveness of interventions. For example, improvements in community and HCWs’ KAP are likely to reduce enacted stigma. Concurrently improving people with TB’s KAP is also expected to mitigate internalised and anticipated stigma. This implies that the ideal and possibly most synergistic intervention would be optimized by delivering interventions to more than one key population. TB clubs, for example, often only benefit people with TB. Stigma-reduction activities and interventions should aim to be more inclusive where possible with HCWS and/or community members being encouraged to attend and participate in TB club meetings, helping to act on all three forms of stigma.

Given that social and economic determinants and consequences of TB are now recognised as significant limiting factors for ending TB, it is notable that there is no recognised national or global indicator for TB-stigma [47]. Currently, there is a global indicator of TB-related catastrophic costs. This indicator has already proven highly useful within research and National TB Programme activities [48] and has led to the creation of a WHO database of the financial burden of TB for more than twenty countries [1]. The same approach should be taken to document the psychosocial burden of TB-stigma across countries. We would strongly advocate for a unified, adaptable global TB-stigma indicator. It could support research and activities to gather data on the prevalence of stigma in different countries or regions. It would also eventually garner further resource investment and scientific interest and heighten much-needed advocacy in this field.

This study has several limitations. First, due to the heterogeneity of study designs, quality, interventions, and tools used, it was not possible to quantitatively determine the effectiveness of interventions. While this was a weakness, the focus was on qualitative assessment of the studies. Second, only articles written in English were included, therefore, some relevant literature may have been missed, particularly of research performed in high TB burden countries in which English is not the first language. Third, paper quality was moderate, and some papers had missing data, which—despite contacting corresponding authors—was not made available for analysis. As our analysis suggested that most papers included were of only low or moderate quality, the power of this review to make conclusions about study impacts is limited [49]. Fourth, we were not able to include studies of complex interventions that aimed to minimise both the psychosocial (e.g. stigma) and economic (e.g. catastrophic costs) of TB, such as the randomised-controlled HRESIPT and CRESIPT studies in Peru [50–52]. While TB treatment, prevention, and economic outcomes from these studies are available, the impact of these interventions on stigma is not yet known. While publication bias is a limitation of any review of the published literature, we attempted to mitigate this bias through a comprehensive grey literature review. Despite the comprehensive evaluation of stigma reduction interventions, this review and framework could not capture the complexity of stigma, which—from a social perspective—involves a set of interrelated, heterogeneous system structures [16].

## Conclusions

Despite the global importance of addressing TB-Stigma, there is a paucity of high-quality studies evaluating interventions to reduce TB-stigma. The novel conceptual framework highlighted that people with TB may experience internalized stigma and anticipated or enacted stigma from three different populations around them: the public, TB HCWs, and other HCWs. Interventions for people with TB can effectively provide psychosocial support for treatment completion. Interventions on HCWs can enhance the support provided for people with TB, and interventions for the public have the potential to reduce community-level stigma toward people with TB. The ideal and possibly most synergistic intervention would be optimized by delivering interventions to more than one key population. Finally, our findings reinforce that it is vital to promote stigma as an indicator in national and international TB strategies to strengthen the development and evaluation of stigma-reducing interventions.

## Supplementary Information

**Additional file 1.** Excluded studies reasoning and Crowe Critical Appraisal Tool (CCAT) Score.

## Data Availability

Data generated or analysed during this study are included in this published article and its additional information files.

## References

[1] World Health Organization (2021). Global tuberculosis report 2021.

[2] Datiko DG, Jerene D, Suarez P (2020). Stigma matters in ending tuberculosis: nationwide survey of stigma in Ethiopia. BMC Public Health.

[3] Teo AKJ, Singh SR, Prem K, Hsu LY, Yi S (2021). Duration and determinants of delayed tuberculosis diagnosis and treatment in high-burden countries: a mixed-methods systematic review and meta-analysis. Respir Res.

[4] Pai M, Kasaeva T, Swaminathan S (2022). COVID-19’s devastating effect on tuberculosis care—a path to recovery. N Engl J Med.

[5] Chapman HJ, Veras-Estévez BA (2021). Lessons learned during the COVID-19 pandemic to strengthen TB Infection control: a rapid review. Glob Health Sci Pract.

[6] Chang SH, Cataldo JK (2014). A systematic review of global cultural variations in knowledge, attitudes and health responses to tuberculosis stigma. Int J Tuberc Lung Dis.

[7] Munro SA, Lewin SA, Smith HJ, Engel ME, Fretheim A, Volmink J (2007). Patient adherence to tuberculosis treatment: a systematic review of qualitative research. PLoS Med.

[8] Courtwright A, Turner AN (2010). Tuberculosis and stigmatization: pathways and interventions. Public Health Rep.

[9] Chowdhury MRK, Rahman MS, Mondal MNI, Sayem A, Billah B (2015). Social impact of stigma regarding tuberculosis hindering adherence to treatment: a cross sectional study involving tuberculosis patients in Rajshahi City, Bangladesh. Jpn J Infect Dis.

[10] Bonadonna LV, Saunders MJ, Zegarra R, Evans C, Alegria-Flores K, Guio H (2017). Why wait? The social determinants underlying tuberculosis diagnostic delay. PLoS ONE.

[11] Craig GM, Daftary A, Engel N, O’Driscoll S, Ioannaki A (2017). Tuberculosis stigma as a social determinant of health: a systematic mapping review of research in low incidence countries. Int J Infect Dis.

[12] World Health Organization (2015). The end TB strategy.

[13] The Global Fund. Tuberculosis and human right. In: The UN General Assembly high-level meeting on ending TB. The Global Fund; 2018.

[14] United Nations (2018). United Nations high-level meeting on the fight against tuberculosis.

[15] Macintyre K, Bakker MI, Bergson S, Bhavaraju R, Bond V, Chikovore J (2017). Defining the research agenda to measure and reduce tuberculosis stigmas. Int J Tuberc Lung Dis.

[16] Pescosolido BA, Martin JK (2015). The stigma complex. Ann Rev Sociol.

[17] Sommerland N, Wouters E, Mitchell EMH, Ngicho M, Redwood L, Masquillier C (2017). Evidence-based interventions to reduce tuberculosis stigma: a systematic review. Int J Tuberc Lung Dis.

[18] Partnership STB (2019). TB stigma assessment: implementation handbook.

[19] Challenge TB. TB stigma measurement guidance. Den Haag: KNCV; 2018.

[20] Hartog K, Hubbard CD, Krouwer AF, Thornicroft G, Kohrt BA, Jordans MJD (2020). Stigma reduction interventions for children and adolescents in low- and middle-income countries: systematic review of intervention strategies. Soc Sci Med.

[21] Crowe M. Crowe Critical Appraisal Tool (CCAT) v 1.4. Queensland; 2013.

[22] Crowe M, Sheppard L (2011). A general critical appraisal tool: an evaluation of construct validity. Int J Nurs Stud.

[23] Thapa S, Hannes K, Cargo M, Buve A, Aro AR, Mathei C (2017). Building a conceptual framework to study the effect of hiv stigma-reduction intervention strategies on HIV test uptake: a scoping review. J Assoc Nurses AIDS Care.

[24] Sommerland N, Masquillier C, Rau A, Engelbrecht M, Kigozi G, Pliakas T (2020). Reducing HIV- and TB-stigma among healthcare co-workers in South Africa: results of a cluster randomised trial. Soc Sci Med.

[25] Idris NA, Zakaria R, Muhamad R, Nik Husain NR, Ishak A, Wan Mohammad WMZ (2020). The effectiveness of tuberculosis education programme in Kelantan, Malaysia on knowledge, attitude, practice and stigma towards tuberculosis among adolescents. Malays J Med Sci.

[26] Bond V, Floyd S, Fenty J, Schaap A, Godfrey-Faussett P, Claassens M (2017). Secondary analysis of tuberculosis stigma data from a cluster randomised trial in Zambia and South Africa (ZAMSTAR). Int J Tuberc Lung Dis.

[27] Croft RP, Croft RA (1999). Knowledge, attitude and practice regarding leprosy and tuberculosis in Bangladesh. Lepr Rev.

[28] Macq J, Solis A, Martinez G, Martiny P (2008). Tackling tuberculosis patients' internalized social stigma through patient centred care: an intervention study in rural Nicaragua. BMC Public Health.

[29] Demissie M, Getahun H, Lindtjørn B (2003). Community tuberculosis care through “TB clubs” in rural North Ethiopia. Soc Sci Med.

[30] Acha J, Sweetland A, Guerra D, Chalco K, Castillo H, Palacios E (2007). Psychosocial support groups for patients with multidrug-resistant tuberculosis: five years of experience. Glob Public Health.

[31] Chalco K, Wu DY, Mestanza L, Muñoz M, Llaro K, Guerra D (2006). Nurses as providers of emotional support to patients with MDR-TB. Int Nurs Rev.

[32] Balogun M, Sekoni A, Meloni ST, Odukoya O, Onajole A, Longe-Peters O (2015). Trained community volunteers improve tuberculosis knowledge and attitudes among adults in a periurban community in southwest Nigeria. Am J Trop Med Hyg.

[33] Wilson JW, Ramos JG, Castillo F, Castellanos EF, Escalante P (2016). Tuberculosis patient and family education through videography in El Salvador. J Clin Tuberc Other Mycobact Dis.

[34] Wu PS, Chou P, Chang NT, Sun WJ, Kuo HS (2009). Assessment of changes in knowledge and stigmatization following tuberculosis training workshops in taiwan. J Formos Med Assoc.

[35] Wouters E, Rau A, Engelbrecht M, Uebel K, Siegel J, Masquillier C (2016). The development and piloting of parallel scales measuring external and internal HIV and tuberculosis stigma among healthcare workers in the Free State Province, South Africa. Clin Infect Dis.

[36] Sommerland N, Wouters E, Mitchell EMH, Ngicho M, Redwood L, Masquillier C (2017). Evidence-based interventions to reduce tuberculosis stigma: a systematic review. Int J Tuberc Lung Dis.

[37] Sengupta S, Banks B, Jonas D, Miles MS, Smith GC (2011). HIV interventions to reduce HIV/AIDS stigma: a systematic review. AIDS Behav.

[38] Bergman A, McNabb K, Farley JE (2021). A systematic review and psychometric appraisal of instruments measuring tuberculosis stigma in Sub-Saharan Africa. Stigma Health.

[39] MacKenzie SB, Podsakoff PM, Podsakoff NP (2011). Construct measurement and validation procedures in MIS and behavioral research: integrating new and existing techniques. MIS Q.

[40] Van Rie A, Sengupta S, Pungrassami P, Balthip Q, Choonuan S, Kasetjaroen Y (2008). Measuring stigma associated with tuberculosis and HIV/AIDS in southern Thailand: exploratory and confirmatory factor analyses of two new scales. Trop Med Int Health.

[41] Koyanagi A, Vancampfort D, Carvalho AF, DeVylder JE, Haro JM, Pizzol D (2017). Depression comorbid with tuberculosis and its impact on health status: cross-sectional analysis of community-based data from 48 low- and middle-income countries. BMC Med.

[42] Naidu T, Pillay SR, Ramlall S, Mthembu SS, Padayatchi N, Burns JK (2020). Major depression and stigma among individuals with multidrug-resistant tuberculosis in South Africa. Am J Trop Med Hyg.

[43] Courtwright A, Turner AN (2010). Tuberculosis and stigmatization: pathways and interventions. Public Health Rep.

[44] Baral SC, Karki DK, Newell JN (2007). Causes of stigma and discrimination associated with tuberculosis in Nepal: a qualitative study. BMC Public Health.

[45] Wouters E, Sommerland N, Masquillier C, Rau A, Engelbrecht M, van Rensburg AJ (2020). Unpacking the dynamics of double stigma: how the HIV-TB co-epidemic alters TB stigma and its management among healthcare workers. BMC Infect Dis.

[46] Rau A, Wouters E, Engelbrecht M, Masquillier C, Uebel K, Kigozi G (2018). Towards a health-enabling working environment—developing and testing interventions to decrease HIV and TB stigma among healthcare workers in the Free State, South Africa: study protocol for a randomised controlled trial. Trials.

[47] Lönnroth K, Glaziou P, Weil D, Floyd K, Uplekar M, Raviglione M (2014). Beyond UHC: monitoring health and social protection coverage in the context of tuberculosis care and prevention. PLoS Med.

[48] Viney K, Islam T, Hoa NB, Morishita F, Lönnroth K (2019). The financial burden of tuberculosis for patients in the Western-Pacific region. Trop Med Infect Dis.

[49] Peters SE, Johnston V, Coppieters MW (2014). Interpreting systematic reviews: looking beyond the all too familiar conclusion. J Hand Ther.

[50] Wingfield T, Tovar MA, Huff D, Boccia D, Montoya R, Ramos E (2017). Socioeconomic support to improve initiation of tuberculosis preventive therapy and increase tuberculosis treatment success in Peru: a household-randomised, controlled evaluation. Lancet.

[51] Wingfield T, Tovar MA, Huff D, Boccia D, Montoya R, Ramos E (2017). A randomized controlled study of socioeconomic support to enhance tuberculosis prevention and treatment, Peru. Bull World Health Organ.

[52] Wingfield T, Tovar MA, Huff D, Boccia D, Montoya R, Ramos E (2016). The economic effects of supporting tuberculosis-affected households in Peru. Eur Respir J.

