# Supplementary material for: Interventions pathways to reduce tuberculosis-related stigma: a literature review and conceptual framework
Source: Infect Dis Poverty. 2022 Sep 23;11:101. doi: 10.1186/s40249-022-01021-8 (PMC9502609; doi:10.1186/s40249-022-01021-8)
Supplement: Supplementary file 1 — Additional file 1. Excluded studies reasoning and Crowe Critical Appraisal Tool (CCAT) Score. [file 40249_2022_1021_MOESM1_ESM.docx]

**Supplements**

Supplement 1 – Excluded studies reasoning

| **Author** | **Year** | **Title** | **Reason for exclusion** |
| --- | --- | --- | --- |
| Kolesnikov, V *et al.* | 1981 | Health education of patients with pulmonary tuberculosis | No intervention |
| Liefooghe, R *et al.* | 1995 | Perception and social consequences of tuberculosis: a focus group study of tuberculosis patients in Sialkot, Pakistan | No intervention |
| Sharp, V *et al.* | 1995 | Implementing a directly observed therapy program in an urban community hospital | Stigma not an outcome measure |
| Dick, J *et al.* | 1996 | Tuberculosis in the community: 2. The perceptions of members of a tuberculosis health team towards a voluntary health worker programme | No intervention |
| Dick, J *et al.* | 1996 | Development of a health education booklet to enhance adherence to tuberculosis treatment | Stigma not an outcome measure |
| Dick, J *et al.* | 1997 | Shared vision--a health education project designed to enhance adherence to anti-tuberculosis treatment | Stigma not an outcome measure |
| Liefooghe, R *et al.* | 1999 | A randomised trial of the impact of counselling on treatment adherence of tuberculosis patients in Sialkot, Pakistan | Stigma not an outcome measure |
| Jaramillo, E | 2001 | The impact of media-based health education on tuberculosis diagnosis in Cali, Colombia | Stigma not an outcome measure |
| Morisky, D.E *et al.* | 2001 | Behavioural interventions for the control of tuberculosis among adolescents | Stigma not an outcome measure |
| Houston, H. R | 2002 | Development of a culturally sensitive educational intervention program to reduce the high incidence of tuberculosis among foreign-born Vietnamese | No intervention |
| Adatu, F *et al.* | 2003 | Implementation of the DOTS strategy for tuberculosis control in rural Kiboga District, Uganda, offering patients the option of treatment supervision in the community, 1998-1999 | Stigma not an outcome measure |
| Dick, J *et al.*, | 2004 | Changing professional practice in tuberculosis care: an educational intervention | Stigma not an outcome measure |
| Mitty, A et al. | 2004 | Community based intervention for marginalised populations | No intervention |
| Clarke, M | 2005 | Towards cost-effective tuberculosis control in the Western Cape of South Africa: intervention study involving lay health workers on agricultural farms | Stigma not an outcome measure |
| Macq, J *et al.* | 2005 | An exploration of the social stigma of tuberculosis in five "municipios" of Nicaragua to reflect on local interventions | No intervention |
| McFarlane, M *et al.* | 2005 | Internet-based health promotion and disease control in the 8 cities: successes, barriers, and future plans | Article was not focussed on TB |
| Rajeswari R *et al.* | 2005 | Perceptions of tuberculosis patients about their physical, mental, and social well-being: a field report from south India | No intervention |
| Rie, A. van *et al.* | 2008 | Measuring stigma associated with tuberculosis and HIV/AIDS in southern Thailand: exploratory and confirmatory factor analyses of two new scales | No intervention |
| Atkins, S *et al.* | 2010 | Patients' experiences of an intervention to support tuberculosis treatment adherence in South Africa | Stigma not an outcome measure |
| Mahmud, N *et al.* | 2010 | A text message-based intervention to bridge the healthcare communication gap in the rural developing world | Article was not focussed on TB |
| Shankar, R *et al.* | 2010 | Perceptions about lung health in Nepal before and after an educational session | Article was not focussed on TB |
| Naidoo, S *et al*. | 2011 | Changes in healthcare workers' knowledge about tuberculosis following a tuberculosis training programme | Stigma not an outcome measure |
| Rocha, C *et al*. | 2011 | The Innovative Socio-economic Interventions Against Tuberculosis (ISIAT) project: an operational assessment | Stigma not an outcome measure |
| Gopu, G *et al*. | 2012 | Impact of health education on the knowledge of tuberculosis among sputum-positive pulmonary TB patients and their caregivers | Stigma not an outcome measure |
| Shen, Z *et al*. | 2013 | Evaluation of health education on TB patients in Pujiang Town of Minhang District of Shanghai City | Stigma not an outcome measure |
| Anand, T *et al*. | 2014 | Perception of stigma towards TB among patients on DOTS & patients attending general OPD in Delhi | No intervention |
| Baral, S *et al*. | 2014 | The importance of providing counselling and financial support to patients receiving treatment for multi-drug resistant TB: mixed-method qualitative and pilot intervention studies' | Stigma not an outcome measure |
| Jadgal, K *et al*. | 2015 | Impact of educational intervention on patient’s behaviour with smear-positive pulmonary tuberculosis: a study using the health belief model | Stigma not an outcome measure |
| Kawatsu, L *et al*. | 2015 | Effect of educational leaflets on knowledge and attitude to tuberculosis among homeless persons in Tokyo, Japan | Stigma not an outcome measure |
| Moya, E *et al*. | 2015 | A project to reduce inequalities and tuberculosis along the US-Mexico border | Stigma not an outcome measure |
| Siegel *et al*. | 2015 | Workplace interventions to reduce HIV and TB stigma among health care workers – Where do we go from here? | Stigma not an outcome measure |
| Tulloch, O *et al*. | 2015 | Patient and community experiences of tuberculosis diagnosis and care within a community-based intervention in Ethiopia: a qualitative study | Stigma not an outcome measure |
| Tola H *et al*. | 2016 | Psychological and educational intervention to improve tuberculosis treatment adherence in Ethiopia based on Health Belief Model: a cluster randomized control trial | Stigma not an outcome measure |
| Khanal, S *et al*. | 2017 | Development of a patient-centred, psychosocial support intervention for multi-drug-resistant tuberculosis (MDR-TB) care in Nepal | No intervention |
| Dsilva, F | 2018 | Exploring workplace TB interventions with foreign-born Latino workers | Stigma not an outcome measure |
| Bisallah, C *et al*. | 2018 | Effectiveness of health education intervention in improving knowledge, attitude, and practices regarding Tuberculosis among HIV patients in General Hospital Minna, Nigeria - A randomized control trial | Stigma not an outcome measure |
| Plowright, A *et al*. | 2018 | Formative evaluation of a training intervention for community health workers in South Africa: A before and after study | Article was not focussed on TB |
| Skiles, M *et al*. | 2018 | Evaluating the impact of social support services on tuberculosis treatment default in Ukraine | Stigma not an outcome measure |
| Sima, B *et al*. | 2019 | Health care providers' knowledge, attitude and perceived stigma regarding tuberculosis in a pastoralist community in Ethiopia: a cross-sectional study | No intervention |
| Ritchie, P *et al*. | 2020 | Impact of peer-trainer leadership style on uptake of a peer-led educational outreach intervention to improve tuberculosis care and outcomes in Malawi: a qualitative study | Stigma not an outcome measure |
| Baniqued, MG *et al.* | 2020 | Social support from nurses and non-adherence with Directly Observed Therapy (DOTS) maintenance phase among patients with tuberculosis in Metro Manila, Philippines | No intervention |
| Nirmal, A *et al.* | 2021 | ‘If not for this support, I would have left the treatment!’; Qualitative study exploring the role of social support on medication adherence among pulmonary tuberculosis patients in Western India | No intervention |
| Yousif, K *et al.* | 2021 | The effect of an educational intervention on awareness of various aspects of pulmonary tuberculosis in patients with the disease | Stigma not an outcome measure |

Supplement 2. CCAT Scores

| **First Author** | **Preliminaries** | **Introduction** | **Design** | **Sampling** | **Data Collection** | **Ethical matters** | **Results** | **Discussion** | **Total** | **Total (%)** | **Low/moderate/high quality** |
| --- | --- | --- | --- | --- | --- | --- | --- | --- | --- | --- | --- |
| **Bond** | 5 | 4 | 4 | 5 | 4 | 4 | 4 | 4 | 34 | **85** | **High** |
| **Idris** | 5 | 4 | 4 | 3 | 5 | 4 | 4 | 4 | 33 | **83** | **High** |
| **Sommerland** | 5 | 5 | 5 | 5 | 5 | 4 | 4 | 5 | 38 | **95** | **High** |
| **Balogun** | 4 | 4 | 3 | 4 | 4 | 4 | 2 | 4 | 29 | **73** | **Moderate** |
| **Wilson** | 4 | 5 | 2 | 1 | 4 | 4 | 2 | 4 | 26 | **65** | **Moderate** |
| **Demissie** | 3 | 5 | 2 | 4 | 4 | 0 | 3 | 3 | 24 | **60** | **Moderate** |
| **Chalco** | 2 | 5 | 2 | 1 | 2 | 3 | 2 | 3 | 20 | **50** | **Moderate** |
| **Acha** | 1 | 3 | 2 | 1 | 3 | 0 | 3 | 3 | 16 | **40** | **Low** |
| **Macq** | 1 | 3 | 2 | 1 | 2 | 2 | 2 | 2 | 15 | **38** | **Low** |
| **Croft** | 2 | 3 | 2 | 1 | 3 | 0 | 2 | 2 | 15 | **38** | **Low** |
| **Wu** | 2 | 4 | 1 | 1 | 2 | 0 | 2 | 3 | 15 | **38** | **Low** |
| ***Average Score*** | *3* | *4* | *3* | *2* | *3* | *2* | *3* | *3* |  |  |  |

All categories scored out of 5. Total score out of 40.
